# Narratives about Negative Healthcare Service Experiences: Reported Events, Positioning, and Normative Discourse of an Active Client

**DOI:** 10.3390/healthcare10122511

**Published:** 2022-12-12

**Authors:** Elina Weiste, Nanette Ranta, Melisa Stevanovic, Henri Nevalainen, Annika Valtonen, Minna Leinonen

**Affiliations:** 1 Finnish Institute of Occupational Health, P.O. Box 40, 00032 Helsinki, Finland; 2 Faculty of Social Sciences (SOC), Tampere University, 33014 Tampere, Finland

**Keywords:** activeness, client’s role, experience, narrative positioning analysis, service satisfaction, service feedback, qualitative research

## Abstract

Narratives about clients’ service experiences in healthcare organizations constitute a crucial way for clients to make sense of their illness, its treatment, and their role in the service process. This is important because the client’s role has recently changed from that of a passive object of care into an active responsible agent. Utilizing Bamberg’s narrative positioning analysis as a method, and 14 thematic interviews of healthcare clients with multiple health-related problems as data, we investigated the expectations of the client’s role in their narratives about negative service experiences. All the narratives addressed the question of the clients’ “activeness” in some way. We identified three narrative types. In the first, the clients actively sought help, but did not receive it; in the second, the clients positioned themselves as helpless and inactive, left without the care they needed; and in the third, the clients argued against having to fight for their care. In all these narrative types, the clients either demonstrated their own activeness or justified their lack of it, which—despite attempts to resist the ideal of an “active client”—ultimately just reinforced it. Attempts to improve service experiences of clients with considerable service needs require a heightened awareness of clients’ moral struggles.

## 1. Introduction

When facing severe chronic illnesses, narratives become essential for coping. Chronic illness constitutes a major “biographical disruption” [1,2] in a person’s life, which threatens the relations between body, mind, social relationships, and everyday habits [3] (p. 31). Narratives are important for re-examining and transforming one’s identity, for maintaining a coherent sense of self, and for giving meaning to the physical, psychological, and social experiences of being ill [1,3,4,5,6]. Illness narratives have been seen as empowering because they may help a person assert control and regain agency in their life [7] (p. 848) and enable the person to gain a “voice” outside the biomedical domain to express their experiential experiences of being ill [8,9,10,11]. 

Narratives about clients’ service experiences in healthcare organizations are also crucial for understanding the ways in which clients make sense of their illness, its treatment, and their role in this [10,12]. For clients with chronic conditions, service narratives can provide a way for them to construct themselves as knowledgeable and experienced service users, who are qualified to evaluate healthcare service systems [12] (pp. 27–30). Clients’ narratives may also contribute to improving the quality of treatment [13]. In positively evaluated narratives, clients have reported actively communicating with healthcare professionals and experiencing trust and appreciation from these professionals [14]. In negatively evaluated narratives, clients have reported being passive when interacting with professionals [14]. They have also experienced a lack of trust and insufficient safety in their client–professional relationships [14].

Clients’ narratives about their service experiences reflect the wider societal and cultural views on the role of a client in healthcare services. Over the past decades, these views have constantly changed. Traditionally, in accordance with the “paternalistic care philosophy”, the role of the client has been rather passive, mainly that of an object of professionals’ procedures and decisions [15,16]. More recently, client-centered views have become guiding paradigms, emphasizing the professionals’ need to understand the clients’ experiences, concerns, and expectations in order to reach a shared understanding of their problem and its treatment [17]. Clients are thus seen as active agents who contribute equally to the collaborative partnership with professionals. They are considered to have crucial experiential knowledge of service processes and to know their service needs best [15]. 

Clients’ and professionals’ expectations regarding the client’s role do not, however, always match. Clients often wish to have a say in service delivery, but they also prefer such involvement to be optional and to vary according to the type and seriousness of their illness and their relationships with professionals [18]. For instance, when the clients’ decision-making power is linked to greater responsibility for their own treatment, clients have shown reluctance to become involved in joint decision-making [19]. When participation is seen as co-determined by clients and professionals, the clients highlight their role in the actual decision-making [18]. Professionals, in turn seem to regard greater client empowerment as threatening professional boundaries and competencies [20]. 

Clients are also noted to differ in the role they take in relation to activities and interactions in their service network [21]. The research on co-creation of value in healthcare services has shown that more active clients seek and share information from a range of sources, connect with people in the service network, redesign their treatment plans, and think positively about their situation [21]. In contrast, more passive clients are considered to accept information given by professionals, comply with recommendations, and collate information, restricting their interactions mainly with their treatment team [21]. The research suggests that the professionals should encourage clients to adopt an active role as it tends to be associated with higher quality of life [21,22].

As noted above, the expectations towards the clients’ role in healthcare services have been studied from multiple viewpoints, considering both the clients’ and professionals’ perspectives (e.g., [15,16,17,18,19,20,21,22]). What has been investigated less is the question how clients themselves address their role in services through their narratives. Our work contributes methodologically and empirically to the discussion on the role of the healthcare clients in four important ways. First, it represents an in-depth qualitative investigation of the healthcare clients’ narratives on their negative service experiences, presenting the client’s first-person perspective on service processes. Second, by utilizing Michael Bamberg’s narrative positioning analysis as a method, it considers narratives not only as expressions of the clients’ experiences or as reflections on what went wrong in a given service-situation but also with reference to their ways of constructing, adopting, and contesting the division of moral obligations and responsibilities between themselves and the professionals. Third, this methodological approach allows us to identify expectations that clients present towards their role, and the role of professionals, in service encounters. Fourth and lastly, our work focuses specifically on clients with multiple, prolonged health-related problems and considerable service needs. By focusing on this specific group of clients, we can give voice to the clients who are most vulnerable but also most knowledgeable in the use of healthcare services, well qualified to evaluate them [12]. 

Our research questions are: (1) how clients position themselves in relation to professionals when talking about their negative services experiences, and (2) what kinds of moral obligations they construct between themselves and professionals in and through their narratives of reported negative events? Our empirical results contribute to the overall understanding of the role of the client in healthcare services and thus ultimately help—we hope—in the identification of those aspects of care that need to be present in good quality services.

## 2. Materials and Methods

### 2.1. Study Setting and Participants

Our dataset consists of 14 thematic interviews of municipal healthcare service users, whom we refer to as “clients”. The interviews were part of the “Social and health care professionals as experts on client involvement” development project of the Finnish Institute of Occupational Health in Finland (FIOH, Helsinki, Finland), which aimed to promote work practices that enhance clients’ involvement in their own care, as well as in the planning and developing of the social and healthcare services. The project organized six regionally comprehensive client-involvement workshops in five different social and health care organizations to develop their organizational work practices. Before the workshop processes, the project’s researchers conducted the interviews in the organizations that were involved in the project. The units were selected to represent different healthcare sectors for adult population, and they consisted of a social and healthcare center, a first-contact service center for elderly people, a rehabilitation ward for people recovering from surgery, an outpatient ward for people undergoing long-term treatment for a chronic condition (such as diabetes), and an outpatient ward for people with mental health problems. 

To deepen our understanding of the clients’ experiences about their role in services, we interviewed clients who regularly used services in at least two different healthcare service sectors and thus had experiential expertise of the service processes. We had no other exclusion criteria for the participation in interviews. The clients were recruited by the healthcare professionals who participated in the project’s workshops. At the time of the interviews, all the clients were undergoing treatment at the organization. Five of the clients were male and nine were female. We have no specific information regarding their age, diagnoses, or other background characteristics, apart from the information that they spontaneously gave us in their interviews.

The interviews were conducted face-to-face in 2019 and early 2020 in the healthcare organizations’ facilities. The length of the interviews varied from 24 to 73 min (11 h of interaction in total). The interviews were audio-recorded and transcribed (total of 257 single-spaced pages).

The themes of the interviews were related to the clients’ perceived participation in their own care and the overall smoothness of the service provision. The clients were asked about their experiences of successful service encounters and problematic service encounters (i.e., “Tell me about a typical service encounter?” or “Could you give me an example of an encounter in which nothing went as you expected?”). These questions prompted the clients to tell narratives about their negative service experiences (see [12]) which were analyzed according to the research questions.

### 2.2. Ethics

The study was conducted in accordance with the Declaration of Helsinki. Permission to collect the data was obtained from each healthcare district and the Ethics Committee of FIOH (23 November 2018). Informed, written consent, including the right to withdraw from the study, was obtained from all participants. The identity of the participants was protected by anonymization and by minimizing any indirectly identifiable data in the publication.

### 2.3. Methods and Analysis

We used narrative positioning analysis to examine how the healthcare service users positioned themselves and the healthcare professionals in their narratives on negative service experiences [23,24,25]. Narrative positioning analysis is based on positioning theory [26,27] and narrative theory [28] and was developed as a theoretical model to explain social actions and the construction of meanings in interpersonal interactions and discursive practices.

Bamberg’s narrative positioning analysis model [23,24,25] analyzes positions as taking place on three levels: (1) the story world, (2) the storytelling situation, and (3) the cultural model stories invoked and drawn upon in the telling [25] (p. 336). In this article, we analyze the positions of the clients and professionals on each of these three levels of the clients’ narratives, focusing on the expectations regarding the client’s role that is constructed through these positionings.

In our qualitative, inductive analysis, we began our analytical process by listening to the recordings of the interviews and reading the transcriptions, making notes on the segments that took a form of a narrative. We collected all the segments in which the topic of the narrative was the client’s own negative service experience. We found 26 such narratives. Thereafter, we started to work with the collection of the narratives in a data-driven way, probing the categories and characters identified in a single narrative against every new narrative in the data. On the story world level, the analysis focuses on how the story characters are positioned in relation to each other, and how the teller positions themselves in relation to the other characters in the story. We analyze the characters of the story (e.g., client, a nurse, a physician) and their relations. On the storytelling situation level, the interest lies in the teller’s positioning in relation to their audience, in our case, the interviewer. Our analysis focuses on the moments of storytelling in which the client invites the interviewer’s response, such as agreement, affiliation, or validation. In this part of the analysis, the conversation analytic method [29,30,31] is applied to examine how the telling was designed to invite a specific type of response from the interviewer. Lastly, the cultural model story level requires observing how both the micro-level identities and cultural identity categories were utilized in this specific institutional context.

In the following Results section, we show and analyze data extracts that represent paradigmatic examples of narrative types found in data. The transcriptions presented in data extracts are simplified and translated from Finnish by authors. The data extracts are chosen from across the dataset in order to demonstrate our findings in a clear, accessible way.

## 3. Results

We found three types of client narratives in our data in which clients positioned themselves differently in terms of the cultural ideal of an “active client”. In the first, most typical narrative type (*n* = 14), the client actively sought help, but did not receive it; in the second, the client positioned themselves as helpless and inactive, left without the care they needed (*n* = 8); and in the third, the client argued against having to fight for care (*n* = 4). In all these narrative types, the clients either demonstrated their own activeness, or justified their lack of it, which—despite attempts to resist the ideal of an “active client”—ultimately merely reinforced it.

### 3.1. Story of a Client Actively Seeking for Help but Not Receiving It

In the first narrative type, the clients position themselves as strong agents who actively seek help. They need clinicians’ professional skills to recognize and inspect their symptoms, and to provide them with correct diagnoses and treatment recommendations. In their narratives, the clients demonstrate that they acted in a morally and normatively correct manner by doing everything in their power to seek help. However, they depict the professionals as incompetent or bureaucratic, and by disregarding their symptoms, as having contributed to the deterioration of the client’s situation, even its acute severity.

Extract 1 exemplifies this narrative. Before the extract, the interviewer has asked the client about her service experience and how it the process could have been smoother. In line 1, the client initiates her response.

**Extract 1.** (C = client, I = interviewer).

01 C: I’ll tell you about such a situation because these

02   pancreas problems of mine began already in twenty fifteen.

03   I regularly had a fever every few weeks

04 I: Yeah.

05 C: so I already said in twenty fifteen to the gastro that

06   I have this fever, I called it a mystery fever.

07   I have a mystery fever so where’s it coming from?

08 I: Yeah,

09 C: So they started really examining my intestine and did

10   an endoscopy, the fever is not because of my intestine.

11   Let’s keep an eye on the situation.

12 I: Yeah.

13 C: I had a bit of a fever now and then and I was off

14   work a lot already then and nowadays too and so on.

15 I: Yeah.

16 C: And the next year I went to the doctor again, I said

17   that because this fever has continued that I’ve been

18   off work so repeatedly

19   just what is really causing this?

20 I: Yeah.

21 C: So the gastro doctor asked has your pancreas

22   been working properly?

23   (0.5)

24 C: And I looked at him like how would I know

25   if it’s working or not?

26   He says have I had backpain.

27   I was like no, and that was it again.

28   Let’s keep an eye on that fever and again the next

29   year there was the different doctor and I talked about this

29   fever and he said that my intestine has been

30   examined it’s not due to the intestine.

31 I: Yeah.

32 C: No one started to look into the fever, no one

33   said let’s give this person a CT scan and

34   that would have shown right away that the pancreas is

35   symptomatic there’s something here.

36 I: Yeah so it dragged on this problem quite

37   significantly?

38 C: Yeah yeah, it dragged on. Then it was the next

39   year and the same continued again. I talked

40   about it,

41 I: Yeah.

42 C: and then umm at the end of the year, week 42 was

43   then, I had last a fever and I wondered about not

44   having a fever for a long time whether it had passed.

45   Because of course I thought that my immunity was

46   so bad because I’d had so many operations and this

47   situation being what it was meant that I couldn’t be

48   particularly active about the issue in any way.

49   I’ve just had these fevers and I’ll have to learn to live

50   ith it I just sort of thought like that.

51 I: Yeah.

52 C: So then it was the beginning of the year when I started

53   becoming slower and slower and slower,

54   one day I couldn’t get from work

55   to the bus station anymore, I almost couldn’t

56   get to my home on the third floor, I almost

57   ended up staying in the stairwell.

58 I: Okay yeah.

59 C: I went to A&E three times. I came here at that time

60   the occupational health center was in (the name of a city)

61   I went there, no one heard how my lungs- the right

62   lung was full of fluid and none of these doctors

63   heard that there was fluid,

64 I: Oh gosh, yes.

65 C: until the fifth on-call doctor said

66   should we scan those lungs? I said that my

67   spirometry score is 180 so please just do something!

68 I: Yeah right.

69 C: Then they were scanned and guess what?

70   What there was in my lung?

71 I: What?

72 C: There was pancreatic fluid and they said

73   that the ducts were so blocked that the pancreas had

74   been symptomatic. The ducts had become blocked so that

75   pancreatic fluid had risen here to my lungs and

76   they took six liters of pancreatic fluid, jet-black

77   fluid out.

78 I: Oh wow, that’s quite.

79 C: And maybe this could have been avoided if someone had,

80   I don’t blame the doctors at all but if someone

81   had thought hey why does this person

82 constantly have a fever,

On the story world level, the main characters in the story are the client and the constantly changing physicians. The story is constructed as a description of subsequent appointments during which the client describes her symptoms and looks for a medical explanation for a “mystery fever” (line 6). The repetition is central for the argumentation: the client refers to the chronologically changing years (lines 02, 16, 28, 39, 42) and numerical quantities (“I went to A&E three times”, line 59; “that fifth on-call doctor said”, line 65) to illustrate her frequent attempts to seek help. Through these attempts, she positions herself as an active and responsible client, knowledgeable enough to grasp that something is wrong. The physicians referred the client for more examinations and asked questions but then decided to merely monitor the situation as nothing alarming was found. Although the client later claims to have known what the physicians should have done (“let’s give this person a CT-scan”, line 33), in the story world, the client needs the physicians’ expertise to get the right kind of help. There are two points in the story at which the physicians ask for the client’s opinion on her condition (lines 21–22 and 65–66). These questions invoke the assumption of an active client who has experiential knowledge and should have a say in her treatment. The client, however, resists this assumption and returns the responsibility to know back to the physician (“how would I know?”, line 24; “please just do something!”, line 67). At the same time, she describes the physicians as incompetent and indifferent to their work. This is evident in lines 61–63, where the client describes a situation in which five physicians have listened to her lungs without noticing that they are “full of black fluid” (line 76). At the end of the story, the client explicitly states that it is the professionals who are responsible for her problematic health outcome (“this could have been avoided if…I don’t blame the doctors at all but…if someone had thought”, line 79–82).

On the storytelling level, the interviewer is invited to validate the story in the moments when the client argues her active attempts to get help. After the description of each help-seeking attempt, the interviewer produces a minimal acknowledgement-token, after which the client concludes her main argument: no one took action to investigate her mysterious symptoms, and the problems in her pancreases remained unnoticed (in lines 32–35). The interviewer formulates the outcome of the client’s telling [32] and acknowledges that the situation was significantly drawn out (lines 36–37). The client confirms the interviewer’s outcome and continues her telling. The interviewer is again invited to participate when the client describes the devastating consequences of her symptoms not being examined and being neglected (“no one heard how my lung was full of fluid”, lines 61–63). The interviewer responds with a Finnish interjection “oho” (“oh gosh”, line 64), which is typically used to convey affectivity such as surprise in response to the previous speaker’s turn [33] (p. 856). In this case, the affective display communicates affiliation with the teller and validates her claim: the situation is so unexpected that its consequences are surprising [34]. Soon after this, the client explicitly invites the interviewer’s response with a prefaced question “guess what, what was there in my lung?” (lines 69–70). Now, the consequence is presented in an even more upgraded and dramatized form: the fluid in the lung is “jet-black” and “six liters” of this fluid was removed from the lungs (lines 75–77). In line 78, the interviewer responds again with a surprise-token “oh wow”, validating and affiliating with the client’s affective stance [35].

On the level of cultural model stories, the client orients towards the ideal of an active client. This idea is not explicitly challenged: in order to receive adequate services, clients need to be active in seeking help. In the story, the client positions herself as a good client who can recognize that something is wrong and who knows how to seek help. She has done everything right and should be entitled to good service. At the same time, she displays trust in the professionals’ knowledge: they should be able to identify the client’s illness and be held responsible for the client’s adequate treatment. In her case, the professionals disregarded their role and the service process failed. The source of the negative experience is thus the professionals’ failure to provide appropriate care, despite the client having acted in a morally and normatively correct manner.

### 3.2. Story of an Inactive, Helpless Client Who Does Not Receive Care

In the second narrative type, the clients position themselves as helpless, unknowing, and inactive, in need of help and left without it. The professionals blame the clients for their inactiveness, even though it is caused by their severe medical condition. A central facet of the narrative is thus providing justification for the clients ending up in a condition which makes activity impossible. Thus, they claim that their inactivity is not their fault and that they would have been entitled to care in their specific situations.

Extract 2 shows an example of this narrative type. Before the extract, the interviewer asked about the client’s experiences of service processes—whether there have been times when everything has worked well and times when the service has failed to meet her expectations. The client responds by stating that she is happy to be alive, after which she initiates her narrative (line 1).

**Extract 2.** (C = client, I = interviewer).

001 C: Well I was in a coma for three weeks and two months

02   in the ICU and I had no pain during that time.

03   They always came to ask if I had any pain and during that

04   month I wasn’t able to talk because I was

05   on a respirator and slightly awake so

06   the little that I was able to communicate with the doctor

07   was to shake my head like this that no,

08   I have no pain.

09 I: Exactly.

10 C: So the pain was gone and well,

11 I: Yeah.

12 C: so it was like shocking that it all happened

13   in one night and it was

14   tough for the psyche of course

15   because at first I didn’t even realize how sick

16   I was and then I began to wake up

17   in the ICU and my daughter said

18   that they have given me a stoma.

19   I started crying at that point because

20   I didn’t understand because I’d only had a small

21   gallbladder attack.

22 I: Yeah exactly.

23 C: So from the beginning I don’t remember anything

24   really because I’d been

25   so disoriented and strong meds and so on but,

26 I: Yeah.

27 C: but well but then there was, if I can tell you one

28   particular example, when I had

29 I: Yeah.

30 C: the stoma then in the beginning and it was uhh it was

31   a stoma that kept coming undone

32   t- this stoma

33   was fixed at some point so that

34   the bag would stay in place better.

35 I: Yes.

36 C: So the nurses blamed me for using up so

37   many stoma bags and I’ll be so expensive.

38 I: Hmh yes,

39 C: And this will stay in my mind forever.

40 I: So it’s quite hurt- has it been quite

41   hurtful then too?

42 C: Well yes, I think it hurt because it wasn’t my fault

43   that this stoma came undone or wouldn’t- I didn’t

44   want to be in this situation just lying down like-

45   well that umm- I couldn’t even change it by myself then

46   so the nurses had to change it because I was

47   in bed for so long that I had to learn

48   to walk and breathe by myself

49 I: Mmh.

50 C: again and to eat and drink and all that.

51   So I couldn’t, I wasn’t able at that time

52   to change the stoma bag so I was like-

53   so I was like told off

54 I: Yeah.

55 C: for being so expensive because I used up

56   the stoma bags as twelve of them could go

57   in one day.

58 I: So exactly the problem was that it-

59   it didn’t stay in place.

On the story world level, the characters in the story are the client, her daughter, a physician, and a nurse(s). The professionals are also referred to with a general plural pronoun “they”. The client positions herself as a teller of the story but describes her agency in the story world as minimal. She is in a coma, undergoes a medical operation, is disorientated, on strong medication, forgetful, disabled, and dependent on the help of the professionals. The professionals are described as distant persons, who come into the room to ask about the client’s symptoms or to perform procedures regardless of what the client wants. These procedures are described as unpleasant for the professionals; the client is accused of neediness and “expensiveness”. Thus, proving her innocence in terms of her state of inactiveness becomes the central part of the story. The client constructs her own innocence in her helplessness by describing how sudden the situation was (“it all happened in one night”, lines 12–13) and she had no opportunity to be prepare for it. The client also describes the severeness of her situation: while in a coma, she had lost her ability to perform even the most taken-for-granted tasks, such as breathing (lines 47–50), let alone changing a stoma bag. The issue was not that she did not want to do something, but that she was unable to do it due to her condition. Moreover, the stoma was applied incorrectly, and kept coming undone (lines 31–34), but the professionals kept blaming her for not being able to deal with this technical problem (lines 36, 42–43). In addition to her descriptions of her weak agency, the client refers to having to relearn to all the basics of life “by herself” (lines 47–50). In other words, no one was there to help or teach her. In and through all this, the client positions herself in the story world as a victim of the professionals’ unfair behavior.

At the level of storytelling, the client builds the story as a response to the interviewer’s question about her experiences with health services. After describing her shock of being in the ward after an extensive medical operation, the client asks for permission to describe “one particular example” of a problematic experience (lines 12–21, 28). Even though the client frames this experience as a single case, it involves the repetition of certain situations: the stoma bag keeps coming undone and needs to be fixed (lines 30–34, 43), and the client is blamed for wasting time and money associated with her care (lines 37, 50–54). After verbalizing these problems, the client turns away from the story world and comments on their significance to the interviewer (“this will stay in my mind forever”, line 39), thus making an affiliative response from the interviewer relevant [35]. The interviewer recognizes the affectivity in the client’s situation and formulates the situation as having been hurtful for the client [36], inviting her confirmation (lines 40–41). The client confirms the interviewer’s formulation and, by circulating the same words, accounts for her experience: it is reasonable to be hurt because she was accused on unfair grounds—of being incapable (lines 40–53) and costly (lines 55–57). At this point, the interviewer confirms the innocence of the client: the problem was not the client, but “the problem was”, the stoma bag that “didn’t stay in place” (lines 58–59). This seems to be the type of response that the client was seeking by recycling the same elements in her story, as after this she moves on to another topic.

On the level of cultural model stories, the client positions herself in the cultural model story that highlights the importance of her own activeness in the treatment processes [15,16]. This model story is apparent in the ways in which the client characterizes the professionals’ expectations of her: the professionals accuse her of being passive and needy. However, the client also resists the ideal of an active client by showing how unreasonable the expectation of activeness can sometimes be. She also invokes a cultural model story according to which society (including healthcare professionals) should take care of its members, specifically when they are weak and vulnerable [37]. She wishes the professionals had recognized her vulnerability and allowed her a role in which she could be taken care of, instead of blaming her for not having been able to fulfil their unreasonable expectations. Nonetheless, paradoxically, the client ends up enforcing the cultural ideal of the “active client”. In casting the professionals’ expectations of her as unreasonable only because of the compelling external circumstance that trumped her desire to be active, she implies that—without such circumstances—it would be reasonable for clients to be active in their own care.

### 3.3. Story of a Competent Client Exerting Influence by Fighting for Care

In the third narrative type, the clients explicitly argue against the idea that the client should fight to receive adequate service. The clients described themselves not only as active clients but also as participating citizens, who give feedback and exert influence when they see problems in service delivery. Extract 3 shows an example of this narrative type. Before the extract, the client told the interviewer that she sometimes feels “left alone” by healthcare professionals. This is followed by the interviewer asking her to give an example of such a situation.

**Extract 3.** (C = client, I = interviewer).

01 C: I’ve been in situations where I’ve really had to kind of

02   fight, for example when the

03   nerve entrapment in my wrist was operated on so I

04   went through a private doctor to

05   (the name of the hospital) back then

06   for the operation and like, they decided there

07   not to operate after all and I

08   demanded that it be operated. I said that the physician had

09   promised me it would be because it affected this shoulder.

10 I: Yeah.

11 C: So well- and it was operated and the effort that it took was

12   stayed on the operating table.

13 I: Yes yes.

14 C: And then, during this operation, it turned out that it

15   had to be done under local anesthetic and then when they

16   started to operate I could feel. It hurt like

17   hell and I had to like dramatize it many times

18   until they realized

19   that it genuinely hurt and then I was anesthetized.

20 I: Exactly yes yes.

21 C: But cases like these that you have to fight for it,

22 I: Yeah

23 C: I think it’s wrong if the person is being operated or

24   treated and no matter how small the thing is

25   you shouldn’t have to fight in that situation

26   for anything that should be done.

27 I: Mm. Mmm.

28 I: Yeah exactly.

((Removed 19 lines of C description of not being heard))

47   I don’t tolerate this sort of thing at all

48   like then I really did I’m quite snappy,

49   I give honest feedback for good and bad and

50   not like rudely but appropriately but then I

51   really sent the issue upwards.

52   Yeah right.

On the story world level, the client frames the story as being about “the fight” between her and the professionals (lines 1–2) and structures it as episodes in which the fighting occurred. The characters in these episodes are the client, a generalized group of professionals (referred to as “they” in lines 6, 15 and 18), and a physician (line 8). In the first episode, the client describes how her wrist operation was about to be cancelled at the last minute (line 7). She demanded that the physician to operate on her, appealing to the referring physician’s promise, which succeeded in making the professionals cancel their decision (lines 8–9). This outcome is presented as a positive one: the operation the client wished for was successful. In the second episode, she experienced pain while under local anesthetic. The client had to “dramatize it many times” until the professionals realized that the anesthetic was not working (lines 17–19). In these episodes, the client positions herself as an active agent whose continuous demands are required for the professionals to act in the right way. She stands up against the bad treatment and defends herself against the professionals. She also takes an evaluative stance towards her own positioning and states that it is wrong and unreasonable that the client has to fight for things to be done during an operation or for correct procedures “no matter how small” (lines 23–26).

On the storytelling level, the client’s story is designed as a response to the interviewer’s request for an example of a negative service experience. The client initiates her story by referring to “situations” she has been in (line 1), founding the story on her real-life experiences. She then uses the phrase “for example” (line 2), accommodating her response to the interviewer’s question. Compared to the previous two data extracts, the interviewer’s responses here are minimal (lines 10, 13, 20, 22, 28) and sequentially placed at moments during which the client describes how she succeeded in receiving better care by demanding it. In this way, the interviewer supports the progress of the client’s telling.

On the level of cultural model stories, the client positions herself as an active, competent agent who is capable and knowledgeable enough to demand the right kind of service from the professionals. Thus, the client not only consistently seeks for help (as in Extract 1) but she also fights to get the services she needs. The client even has to “dramatize” her pain to make the professionals realize that she is really hurting (line 18). She also explicitly criticizes the idea that the client has to fight to get things done right—even in the middle of an operation (line 23–26). She further justifies how she behaves in these situations: she “won’t tolerate this kind of thing at all” (line 47) and is “quite snappy and gives honest feedback” (lines 48–49). She also positions herself as one not in search of a fight and gives feedback “not rudely but appropriately” (line 50). By this, she positions herself as a person who is acting normatively correctly and who is not responsible for the failures in the situation. Thus, in this case, the master narrative is not only about the ideal of an active client but also about an agentive citizen who gives feedback and exerts influence to build a fairer society and to ensure smoother delivery of services (e.g., [38]).

## 4. Discussion

In this study, we explored the ways in which clients construct their role as a responsible agent in clients’ narratives about their negative services experiences. The analysis extends prior research which has mainly studied the contents of clients’ negative service experiences [13,14] or activities the client are engaged when they participate in service processes [21,22]. Our findings showed that the clients with chronic conditions oriented towards cultural model stories which highlighted the importance of the client’s activeness in their service processes (e.g., [15,16,17,18,19]).

We found three types of narratives. In the first, the clients actively sought help but did not receive it; in the second, the clients were positioned as helpless and inactive, left without the care they needed; and in the third, the clients argued against having to fight for care. In all these narrative types, the clients either demonstrated their own activeness, or justified their lack of it, which, despite attempts to resist the ideal of an “active client”—ultimately only reinforced it. These contradicting views may reflect the wider societal and cultural change in how the role of a client is perceived in healthcare services [15,16], and illustrate how difficult it is to resist the prevailing cultural model stories.

The clients’ activeness was addressed in all the negative service stories in our dataset (cf., [14]). However, the source of the negative experience differed in the three types of narratives. In the first, it was the professionals’ failure to provide the right kind of care, regardless of the client being active in seeking help. In the second type of narrative, the source of the negative experience was the professionals’ unreasonable expectations regarding the client’s activeness, and the lack of the care that the client wished to receive. In the third, the client complained about having to fight in order to be heard by the professionals. These negative experiences show that the expectations of the role of an active agent may be quite challenging for clients. This is especially the case when clients have lost their ability to be active, due to the very same reasons that they have sought the healthcare service in the first place. The clients have to be ill enough to be given the healthcare service, yet competent enough to mind the active client master narrative. In our dataset, the most extreme case was in an intensive care unit (Extract 2), in which clients are inherently vulnerable—on one hand, because of their critical illness and their need for intensive care and, on the other hand, due to a lack of the main human characteristics: consciousness, agency, and self-determination [39]. Consequently, critically ill clients may lose control over their situation, falling into total dependence on the healthcare team [40] (p. 747). The requirements of activeness thus come across as entirely unfair, our study highlighted a further aspect in this unfairness—the clients having to engage in extra interactional work to justify their experiences and thus prove their innocence in the face of the dominant, yet remarkably one-sided, cultural ideal of the “active client”. This constitutes a “double burden” to people who have already faced difficult experiences by mere virtue of their illness.

Our analysis highlights the importance of narratives in understanding experiences of healthcare. Narratives, viewed as a form of social practice, afford the opportunity to study how individuals make sense of their experiences of illness and social processes in service-systems [41]. Bamberg’s [23,24,25] three-level analysis of narrative positioning provided analytical tools for examining the construction of narratives in situ [42]. By analyzing not only the contents of their stories but also the ways in which the story was told to the interviewer, we gained an insight into the emotionally heightened moments that the clients experienced as challenging. The clients seemed to invite the interviewer to respond and validate their experience during the moments when they revealed the core of their narrative, such as the challenging experience of having been left without treatment regardless of their active attempts to seek it (Extract 1) or being wrongly accused of inactivity when having lost their basic human functions (Extract 2). The analysis of narrative positioning thus helped us grasp the meaning of the narratives to the clients themselves.

Although the strength of our study is in the detailed three-level analysis of healthcare clients’ narratives, it also has certain limitations. An obvious limitation is the relatively small number of participants in our data, which constrains the generalizability of our results. Healthcare clients are, of course, a very heterogeneous group of people with different needs, expectations, and resources for participating in their care [18]. The clients in this study represented a specifically disadvantaged sample, as they all had multiple, prolonged health-related problems and considerable service needs, which substantially affected their everyday functioning, but then again, they may also be considered to be specifically experienced clients with massive expertise in being in a role of the client in different services. We do not know if those clients who are in good health and only occasionally need services would experience their role differently. One may also ask how the specific service sector (e.g., internal medicine, elderly care, psychiatry) influences clients’ narratives. Based on our data, however, the negative service experiences did not essentially differ in the different healthcare service sectors. In contrast, in our data, all the three narrative types were equally represented, regardless of the service sector. However, possible differences between client groups with diverging service needs remain a topic for future research.

Identifying and describing different ways of experiencing healthcare service quality provides professionals with a strategic insight into clients’ first-person perspectives of service encounters. This type of experiential knowledge cannot be obtained through client satisfaction questionnaires [43]. As our analysis shows, the source of the actual dissatisfaction was not only the negative event as such (e.g., an inappropriate treatment decision or an impolite interaction), but the normative expectations of the clients and of the professionals’ roles in the service encounters, and most importantly, their mismatch, which seemed to underlie the great moral struggle as the clients—in their narratives—sought to present themselves as morally worthy. Hence, all attempts to improve clients’ service experiences first require a heightened awareness of clients’ moral struggles: only on this basis can healthcare professionals find ways to genuinely improve the quality of their professional practices.

## 5. Conclusions

In terms of content, the healthcare clients’ negative service experiences emphasized three aspects that are important for good-quality healthcare: easy access to services, appropriate diagnosis and care that meets the client’s needs, and respectful empathetic interaction. The narrative analysis of the story world level revealed that under the surface of the content is a further source of negative experiences, which is anchored in the moral and cultural ideals and expectations that the client must be active. Through their narratives, the clients adopted and contested the moral obligations related to these expectations and allocated the responsibilities between the professionals and themselves.

For interview research, the analysis of the storytelling level is a viable option to consider. Often the analysis focuses only on the content of the interviewee’s talk without considerations of what is happening in situ between the interviewer and interviewee. Our analysis shows that the storytelling level may help researchers to grasp the meaning that the talk has to the interviewee themself and thus to strengthen their first-person perspective in research.

The recent cultural change from paternalistic care philosophy to the present notions of client involvement have perhaps made professionals emphasize only the advantages of the clients’ activeness in the care process. Moreover, prior research has supported this viewpoint advising professionals to encourage clients to adopt an active role as it may engender better quality of life [21,22]. Often, the clients themselves wish to be actively involved in the service delivery [18]. However, they also want professionals to recognize this involvement as optional and as varying according to the context, time, and individual situation of the client [18]. Considering the strain that severely ill healthcare clients are under when they need care, unreasonable expectations of their activeness may be an extra burden and the source of a negative service experience. Thus, more awareness is needed of the cultural expectations regarding the client’s role in clinical encounters. As the clients orient towards these expectations anyway, they cannot be ignored. Instead, good-quality care must involve an active, explicit strategy to minimize the burden that these expectations may sometime cause clients.

## Data Availability

Available on request in Finnish.
